# Parathyroid carcinoma: an unusual presentation of a rare neoplasm

**DOI:** 10.3205/000262

**Published:** 2017-12-28

**Authors:** Sharma Shruti, Fouzia Siraj

**Affiliations:** 1 National Institute of Pathology, ICMR, Safdarjung Hospital Campus, New Delhi, India

**Keywords:** hyperparathyroidism, parathyroid adenoma, parathyroid carcinoma

## Abstract

Parathyroid carcinoma is an extremely rare malignant endocrine neoplasm that is very challenging in its diagnosis as well as its treatment. Clinically the disease is detected earlier in patients who present with hyperparathyroidism with signs of profound hypercalcemia. Differentiation between benign and malignant disease of the parathyroid is challenging both for the clinician and for the pathologist. Complete surgical resection at the time of first operation offers the best chance of cure. Even after radical excision which is the standard management, local recurrence and metastases are frequent. The disease usually has a slow indolent course and most patients suffer from complications of hypercalcemia rather than tumor invasion or metastasis.

We report a case of a 31-year-old woman who presented with renal colic. Various hematological, biochemical and radiological investigations were performed and a slightly enlarged right parathyroid was found. A clinical diagnosis of parathyroid adenoma was made and a right parathyroidectomy was done. Intraoperatively the surgeon had no suspicion of malignancy but microscopically the lesion was malignant and a final diagnosis of parathyroid carcinoma was rendered based on the criteria of invasion.

Since there is no gold standard, a multidisciplinary approach, including the entire clinical, biochemical, radiological and pathological profile of the disease aids in an accurate diagnosis. Here we are reporting a case of a functional parathyroid carcinoma presenting in a relatively young patient with all the biochemical and radiological investigations and intraoperative findings pointing towards a benign parathyroid disease.

## Zusammenfassung

Das Nebenschilddrüsenkarzinom ist eine extrem seltene, bösartige endokrine Neoplasie, deren Diagnose und Behandlung eine große Herausforderung darstellen. Klinisch wird die Erkrankung früher in Patienten mit Hyperparathyreoidismus mit Zeichen von schwerer Hyperkalzämie entdeckt. Die Unterscheidung von gutartigen und bösartigen Erkrankungen der Nebenschilddrüse ist sowohl für den Kliniker als auch für den Pathologen schwierig. Eine komplette operative Entfernung bei der ersten Operation bietet die beste Chance auf Heilung. Sogar nach radikaler Entfernung, was die Standardbehandlung darstellt, treten häufig lokale Rekurrenzen und Metastasen auf. Die Erkrankung hat normalerweise einen langsamen und schmerzfreien Verlauf und die meisten Patienten leider vorrangig unter Beschwerden, die von der Hyperkalzämie herrühren und weniger von der Ausbreitung des Tumors oder der Metastasen.

Wir berichten von dem Fall einer 31-jährigen Frau, die unter einer Nierenkolik litt. Verschiedene hämatologische, biochemische und radiologische Untersuchungen wurden durchgeführt, wobei eine leicht vergrößerte rechte Nebenschilddrüse festgestellt wurde. Ein Nebenschilddrüsenadenom wurde diagnostiziert und die rechte Nebenschilddrüse entfernt. Während der Operation bestand kein Verdacht auf Bösartigkeit. Eine mikroskopische Untersuchung zeigte jedoch, dass die Läsion bösartig war, woraufhin die endgültige Diagnose aufgrund der Tumorinvasion Nebenschilddrüsenkarzinom lautete.

Da es keinen Goldstandard gibt, hilft ein multidisziplinärer Ansatz, der alle klinischen, biochemischen, radiologischen und pathologischen Instrumente der Diagnose einschließt. Wir berichten hier über den Fall einer relativ jungen Patientin mit funktionellem Nebenschilddrüsenkarzinom, bei dem alle biochemischen und radiologischen Untersuchungen sowie intraoperativen Befunde zunächst auf eine gutartige Nebenschilddrüsenerkrankung hinwiesen.

## Introduction

Parathyroid carcinoma (PCA), a rare endocrine malignancy first described by de Quervain in 1904, occurs either sporadically or as a part of a genetic syndrome [1]. It accounts for less than 1% cases of sporadic primary hyperparathyroidism worldwide with a higher incidence of 5% in Japan and Italy. It affects men and women equally usually in the fourth or fifth decade of life [2]. Prior neck radiation, end-stage renal disease or occurrences of an adenoma or a hyperplastic parathyroid gland (PT) have been associated with an increased incidence of PCA [3], [4]. Majority (90%) of these tumors are functional hormone-producing with elevated serum parathyroid hormone and calcium levels. Symptoms of hypercalcemia are also seen in various benign causes of hyperparathyroidism thereby causing difficulty in diagnosing PCA preoperatively [1]. Nonfunctional tumors have also been reported and are found to be more aggressive than their functional counterparts [5], [6]. These neoplasms are usually solitary with an indolent but slowly progressive course [2].

We herein report a case of a young female patient who presented clinically with features of hyperparathyroidism. 

## Case report

**Figure 1:**
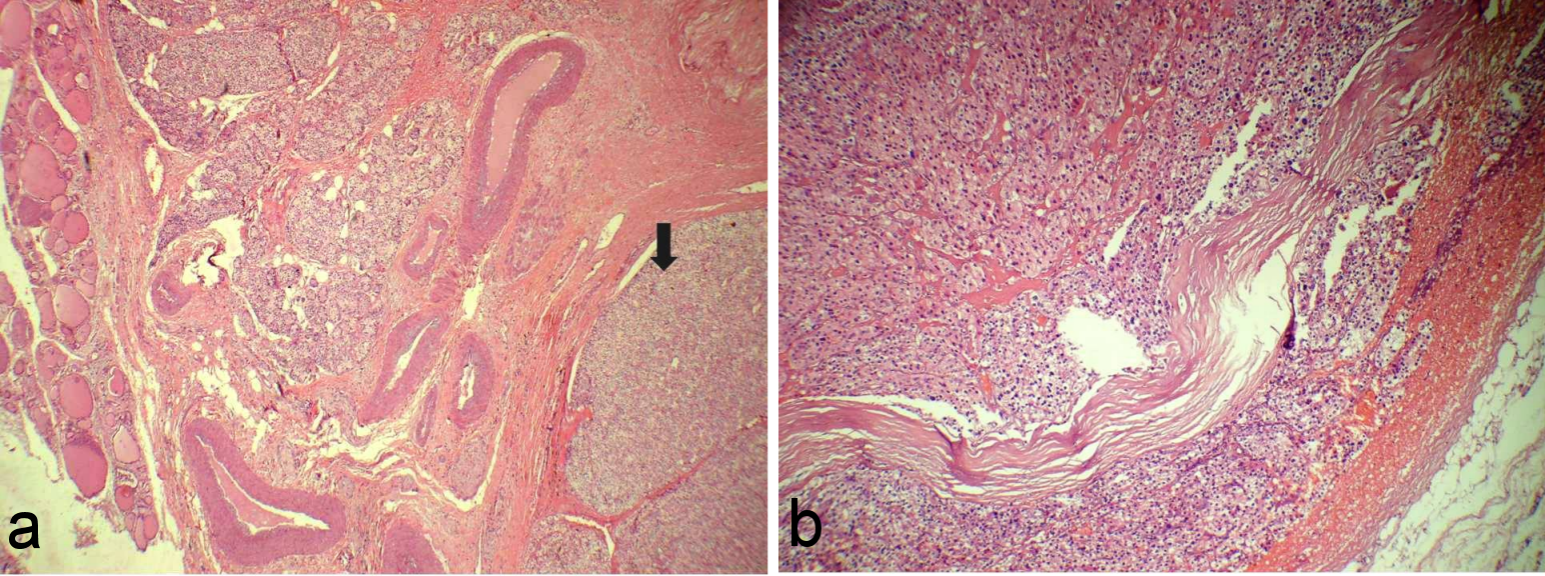
a: Photomicrograph shows an encapsulated parathyroid tumor (arrow) along with normal thyroid and parathyroid tissue (H&E 100X). b: Photomicrograph shows fibrous bands dividing the neoplasm into lobules with cells arranged in sheets and trabeculae (H&E 100X).

**Figure 2:**
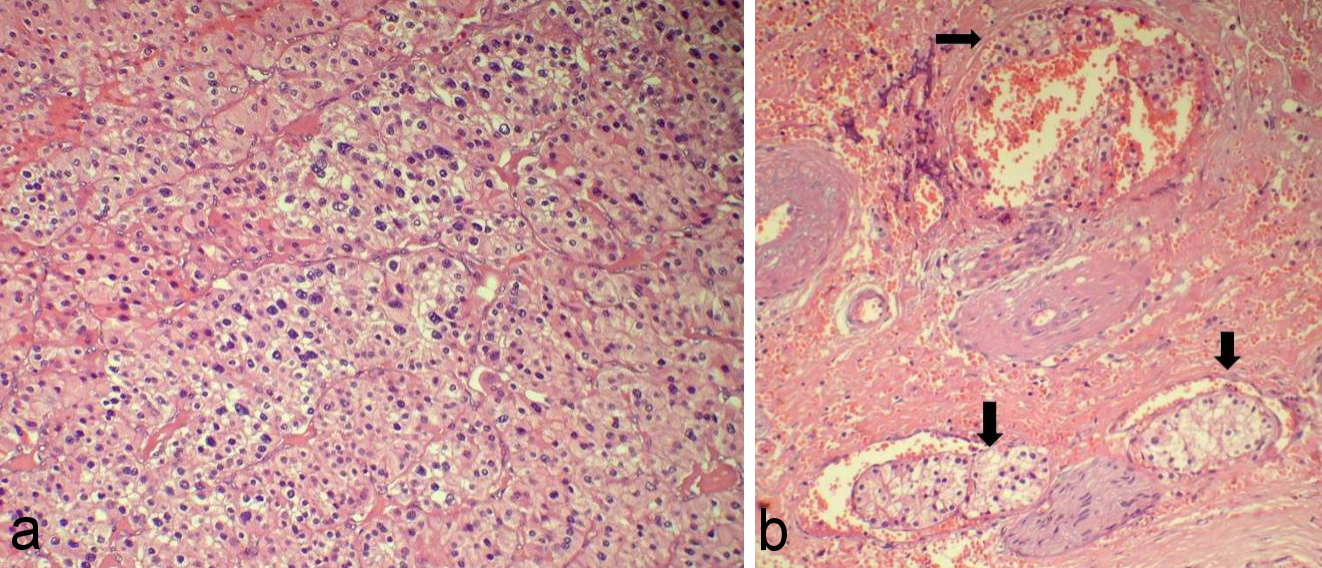
a: Photomicrograph shows medium to large cells with round to ovoid nuclei with prominent nucleoli and abundant eosinophilic, granular to clear cytoplasm (H&E 200X). b: Photomicrograph shows vascular invasion with presence of numerous vascular emboli at the periphery of the tumor (arrows) (H&E 200X).

A 31-year-old woman presented to the emergency department with renal colic. She also complained of nausea, vomiting, heart burns and persistent pain in the back and legs for several months. Routine hematological investigations revealed a slight elevation of the serum calcium levels to 11 mg/dl and serum creatinine to 1.7 mg/dl. In view of the raised serum creatinine, calcium and persistent bone pain, a serum parathyroid hormone assay was performed and a raised value of 148 pg/ml was obtained. On clinical examination no neck mass was found. Ultrasonography and 99mTc-sestamibi scintigraphy of the neck revealed only a slightly enlarged right PT. With a clinical diagnosis of parathyroid adenoma a right parathyroidectomy was performed. Macroscopically, the PT was reddish-brown, soft to firm, measured 2 cm and weighed 2.5 g. Cut section revealed a 1 cm whitish zone which appeared fibrous with hemorrhagic areas. Microscopic examination showed normal thyroid and an encapsulated tumor in the parathyroid gland (Figure 1a (Fig. 1)). Multiple fibrous bands extended from the thickened capsule dividing the neoplasm into lobules with cells arranged in sheets and trabeculae (Figure 1b (Fig. 1)). The cells were medium to large, had round to ovoid nuclei with prominent nucleoli and abundant eosinophilic, granular to clear cytoplasm (Figure 2a (Fig. 2)). Nuclear pleomorphism was seen along with mitotic activity. Both capsular and vascular invasion were seen at the periphery of the neoplasm (Figure 2b (Fig. 2)). Final diagnosis of PCA was based on the criteria of invasion. 

Postoperatively on the second day serum calcium and serum PTH levels reduced to 8.5 mg/dl and 30 pg/dl respectively. On follow-up at 3, 6, 9 and 12 months the patient was disease-free with normal serum PTH and calcium and a negative whole body positron emission tomography scan one year after surgery. The patient was disease free for 1 year and after that has not returned for follow-up. 

## Discussion

Due to the lack of differentiating features specific for PCA, utmost importance is given to distinguishing a malignant lesion from a benign disease before surgery. Some clinical and pathological differences between a parathyroid adenoma and a PCA have been summarized in Table 1 (Tab. 1). PCAs are usually diagnosed in patients between the ages of 45 and 60 years, a decade younger than patients with an adenoma [7]. While benign parathyroid diseases are more common in females in a ratio of 3–4:1, PCA does not show any sex predominance [2]. Though 30–76% patients of PCA present with a palpable mass in the neck, fewer than 5% of patients with benign disease have a palpable neck mass [2]. Majority of PCA (>90%) are functional with markedly elevated serum parathyroid hormone usually up to 40 times and calcium levels of more than 14 mg/dl. This leads to symptoms specific of hypercalcemia raising a suspicion of parathyroid carcinoma. Parathyroid hormone levels in adenomas typically don’t exceed three times the normal upper limit [5]. 

The signs and symptoms of hypercalcemia dominate the clinical picture with typical hyperparathyroid bone disease and renal involvement [7]. PTH levels greater than 300 pg/ml indicate a potentially malignant disease with clinical manifestations of hyperparathyroidism appearing much before local invasion of tumor. The patients experience fatigue, nausea, vomiting, anorexia, dyspepsia, loss of weight and appetite, headaches, polydipsia, polyuria, pathological fractures, muscular pain, and renal diseases like nephrolithiasis or nephrocalcinosis in severe hyperparathyroid states [3], [4]. 

Imaging techniques such as ultrasonography, 99mTc sestamibi scan and tomographic examination of the neck help in localizing and defining the extent of the neck mass but do not help in assessing the malignant potential [1], [2]. MIBI scintigraphy is 91% sensitive in assessing the presence and localization of PCA [2]. Differential diagnosis includes thyroid carcinoma and benign parathyroid disease [2]. Fine needle aspiration is not conducted due to the possibility of tumor seeding from the needle track and difficulty in distinguishing benign from malignant lesions on cytology [1].

Altered expression of various oncogenes and tumor suppressor genes namely p53, breast carcinoma susceptibility (BRCA2), cyclin D1, parathyroid adenomatosis gene 1 (PRAD1), retinoblastoma (RB), and hyperparathyroidism 2 (HRPT2) tumor suppressor genes have been implicated in the pathogenesis of PCA. Shattuck et al. have documented HPRT2 mutation in 10 of 15 patients with sporadic PCA [2]. HPRT2 encodes a tumor suppressor protein parafibromin which inhibits cell proliferation by blocking cyclin D1. Loss of parafibromin expression leads to over expression of cyclin D1 leading to tumor growth. In a study by Vassef et al. cyclin D1 over expression was observed in 91% (10/11) of PCA specimens as compared to 39% (11/28) of the adenoma specimens [4], [7].

Differentiating a PCA from an adenoma is challenging for the pathologist. Grossly, parathyroid carcinomas can be indistinguishable from adenomas as in our case though they usually present as larger (>3 cm), firm, lobulated, grey-white masses weighing 2–10 g, with a dense, fibrous capsule adherent to adjacent structures. Adenomas in contrast are round to oval, reddish-brown in colour and of a soft consistency [3], [4]. Due to the absence of these gross findings in our case there was no suspicion of a carcinoma at the time of surgery. Microscopically monomorphic cells, diffusely enlarged nuclei with macronucleoli and presence of thick intratumoral fibrous bands are suggestive but not diagnostic of malignancy. Pleomorphism is not diagnostic of malignancy as focal pleomorphism is also commonly seen in adenomas [5]. Focal calcification, cystic changes, coagulative necrosis and mitotic figures are commonly appreciated [4]. Malignancy is confirmed on the basis of either vascular or capsular invasion with growth into adjacent structures or by distant metastases [4]. Entrapped tumor cells in the fibrous capsule can be mistaken for invasiveness but is not a sign of malignancy [5]. A triad of macronucleoli, greater than five mitoses per 50 high power fields and presence of necrosis correlates with an aggressive and recurrent disease [5]. The foremost differential is parathyroid adenoma followed by parathyroid hyperplasia. Possibility of anaplastic carcinoma of thyroid and metastatic renal cell carcinoma must also be excluded [8]. Immunoperoxidase staining to exclude thyroid origin is obligatory [5]. Due to these practical issues various researchers have shown keen interest in developing other methods such as immunohistochemistry and DNA analysis but there is no single marker sensitive and specific enough to diagnose PCA [4]. A recent study by Frenandez-Ranvier et al. has proposed a reliable combination of loss of parafibromin with overexpression of RB and galectin-3 in differentiating PCA from atypical adenoma and other benign lesions [4].

 Since PCA is a slow-growing tumor with distant metastases occurring late in the disease course, aggressive surgical approach is recommended to avoid local recurrence [7]. Complete surgical en bloc tumor resection with microscopically negative margins is the treatment of choice for PCA and should be performed with ipsilateral thyroidectomy to avoid any capsular rupture [2]. Surgeons avoid intraoperative biopsies to preserve the capsular integrity and prevent spillage of tumor cells, which is of utmost importance [5]. Monitoring PTH levels intraoperatively helps in predicting the cure rate. A significant drop (>50%) reflects an optimal surgery in en bloc resection of the tumor [2]. Elevated values could be due to a residual tumor in the neck or some undetected metastatic foci [4]. The situation becomes complex when the diagnosis is made on pathology as in our case with presence of extensive vascular or capsular invasion. If the patient remains in remission after surgery as seen in this case under discussion, immediate reoperation may not be required as complete resection of the tumor is usually curative. These patients should be observed carefully with frequent monitoring of their PTH and serum calcium levels [3]. Development of symptomatic hypocalcaemia by virtue of calcium and phosphorus being deposited into the bones is regarded as a sign of successful surgery [4].

Recurrent disease occurs in more than 50% patients of PCA [1]. Metastasis occurs via lymphatic and hematogenous routes commonly to the regional lymph nodes, lungs, bones and liver [4]. Recurrent or metastatic PCA are managed surgically with wide resection including the regional lymph nodes resulting in normocalcaemia for months to years [1], [2]. Typically, patients of PCA require repeated surgeries predisposing them to surgery-related risks with each operation. The efficacy of chemotherapy and radiotherapy in eradication of PCA is controversial. Since PCAs are not considered radiosensitive, radiotherapy is ineffective either as monotherapy or as an adjuvant to surgery [2], [3]. Few studies however suggest radiotherapy as a useful adjuvant therapy in preventing recurrence after surgery [6]. Though there is no consensus in the literature regarding the doses to be administered, these studies report doses ranging from 50 to 66 Gy with no recurrences 2 and 4 years after surgery. The average period of recurrence is usually 3 years but longer intervals of up to 20 years have been reported in literature [2], [4]. For inoperable widely disseminated PCA a relatively prolonged survival is possible by controlling hypercalcemia with calcimimetics [4]. The majority of patients eventually suffer from complications of hypercalcemia like renal failure, cardiac arrhythmias or pancreatitis rather than from tumor burden [1].

Survival rates vary from 90% at 5 years to 67% at 10 years in patients who undergo complete en bloc tumor resection [4], [8]. Lymph node metastases at the time of diagnosis, distant metastases and non-functioning carcinomas are a few negative prognostic factors [4]. 

## Conclusion

PCA is a very rare disease with a delayed diagnosis especially when the patient has non-specific symptoms, the tumor is non-palpable and the serum calcium and parathyroid hormone are not too high. In this case neither the clinical symptoms nor the serum calcium and parathyroid hormone levels indicated severe hyperparathyroidism as usually seen in PCA. The patient’s young age for carcinoma was also quite unusual. Moreover, the preoperative localizing studies and intraoperative gross evaluation did not raise a suspicion of the presence of a parathyroid carcinoma. Postoperatively, only the histopathological examination of the clinically suspected adenoma revealed a diagnosis of PCA. Even the gross findings of the resected parathyroid favored an adenoma. 

For the clinician challenge lies in differentiating hyperparathyroidism caused either by a PCA or its benign counterpart which is much more common. Patients of PCA with persistent hypercalcemia should undergo thorough investigations with a lifetime follow-up since recurrences upto 20 years have been reported [2]. Considering PCA in the differential diagnosis of hypercalcemia due to raised PTH is of utmost importance because complete resection of the tumor is essential at the time of the initial diagnosis else it would lead to substantial morbidity and mortality [3]. Due to very low incidence of PCA, no study trials have been conducted on the efficacy of radiotherapy and chemotherapy [4]. The best practice would probably be to approach each patient individually in a multidisciplinary fashion. PCA is an aggressive tumor with probability of multiple recurrences that portends a poor outcome [7].

## Notes

### Competing interests

The authors declare that they have no competing interests.

## Figures and Tables

**Table 1:**
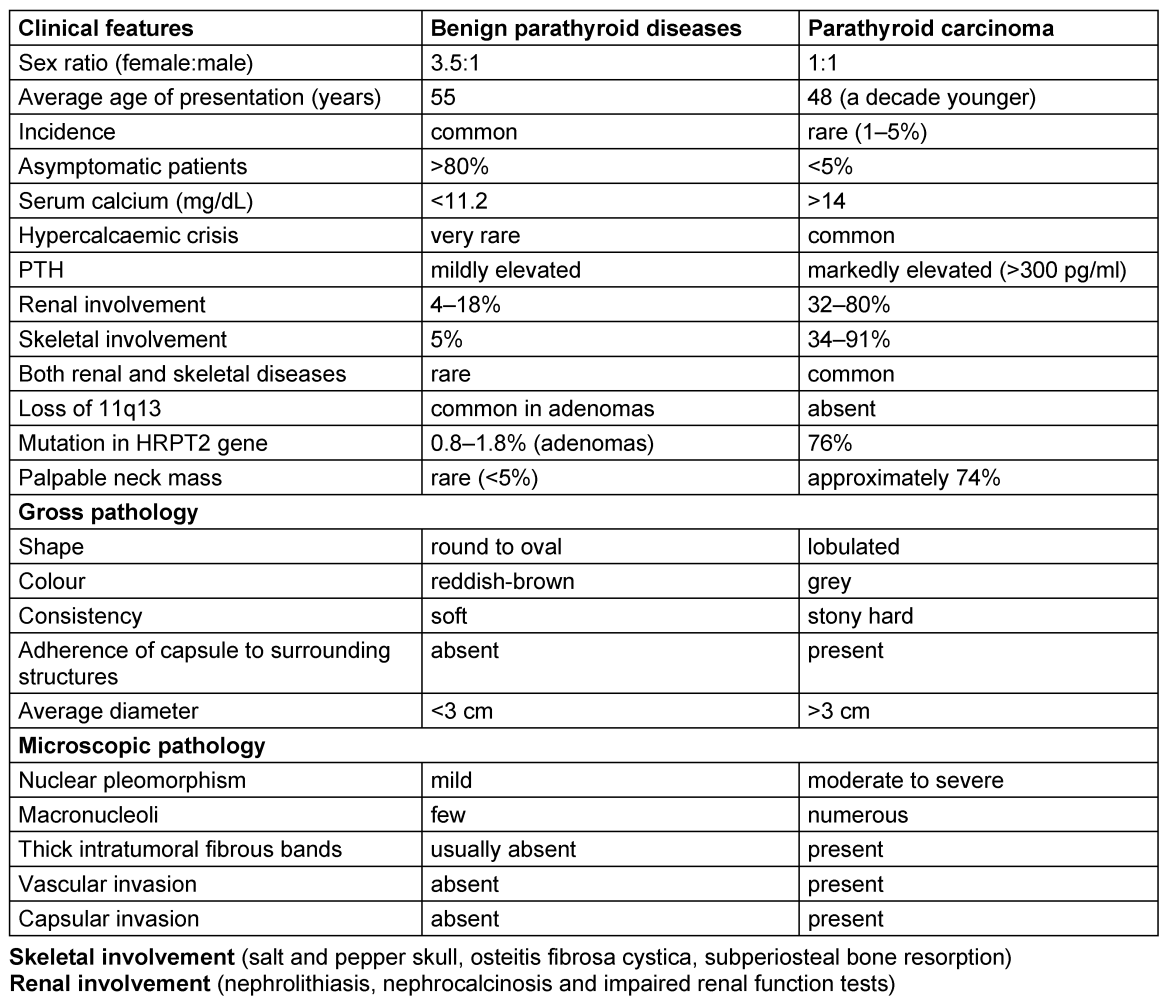
Clinical and pathological differences between a benign parathyroid disease and a parathyroid carcinoma
